# Di-μ-benzoato-κ^3^ *O*,*O*′:*O*′;κ^3^ *O*:*O*,*O*′-bis[aqua­bis­(benzoato-κ^2^ *O*,*O*′)(dimethylformamide-κ*O*)europium(III)]

**DOI:** 10.1107/S1600536811042735

**Published:** 2011-10-22

**Authors:** Zhiliang Wang, Xianju Zhou

**Affiliations:** a College of Optoelectronic Engineering, Chongqing University of Post and Telecommunications, Chongqing 400065, People’s Republic of China; b Department of Mathematics and Physics, Chongqing University of Post and Telecommunications, Chongqing 400065, People’s Republic of China

## Abstract

The title dimeric complex, [Eu_2_(C_7_H_5_O_2_)_6_(C_3_H_7_NO)_2_(H_2_O)_2_], is centrosymmetric, implying that pairs of equivalent Eu^3+^ ions and ligands lie *trans* to each other and that the two Eu^3+^ ions have exactly the same coordination environment. Each Eu^3+^ ion is nine-coordinated by two bidentate benzoate ligands, two bridging tridentate chelating benzoate ligands, and one dimethylformamide and one water molecule. The coordination polyhedron of each Eu^3+^ ion can be described with a distorted monocapped square-anti­prismatic geometry. The mol­ecular structure is stabilized by intra- and inter­molecular hydrogen bonds between the water mol­ecules and benzoate O atoms.

## Related literature

For properties of rare earth compounds derived from carboxylic acids, see: Chin *et al.* (1994 ▶); Singh *et al.* (2002 ▶). For related compounds, see: Jin *et al.* (1996 ▶); Gubina *et al.* (2000 ▶); Wang *et al.* (2003 ▶); Qiu *et al.* (2007 ▶); Ooi *et al.* (2010 ▶).
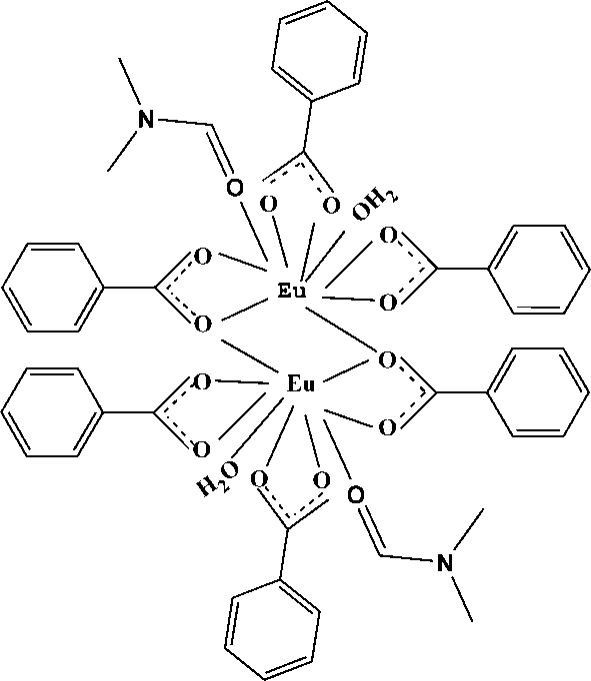


## Experimental

### 

#### Crystal data

[Eu_2_(C_7_H_5_O_2_)_6_(C_3_H_7_NO)_2_(H_2_O)_2_]
*M* *_r_* = 1212.80
Monoclinic, 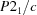

*a* = 11.6395 (2) Å
*b* = 8.3692 (1) Å
*c* = 25.5235 (4) Å
β = 101.460 (2)°
*V* = 2436.76 (6) Å^3^
*Z* = 2
Cu *K*α radiation
μ = 18.84 mm^−1^
*T* = 291 K
0.36 × 0.32 × 0.32 mm

#### Data collection

Oxford Gemini S Ultra diffractometer
Absorption correction: multi-scan (*CrysAlis PRO*; Oxford Diffraction, 2010 ▶) *T*_min_ = 0.056, *T*_max_ = 0.065
8782 measured reflections
4294 independent reflections
3860 reflections with *I* > 2σ(*I*)
*R*_int_ = 0.035

#### Refinement

*R*[*F* ^2^ > 2σ(*F* ^2^)] = 0.045
*wR*(*F* ^2^) = 0.120
*S* = 1.03
4294 reflections
315 parameters
3 restraints
H atoms treated by a mixture of independent and constrained refinement
Δρ_max_ = 1.20 e Å^−3^
Δρ_min_ = −1.92 e Å^−3^

### 

Data collection: *CrysAlis PRO* (Oxford Diffraction, 2010 ▶); cell refinement: *CrysAlis PRO*; data reduction: *CrysAlis PRO*; program(s) used to solve structure: *SHELXS97* (Sheldrick, 2008 ▶); program(s) used to refine structure: *SHELXL97* (Sheldrick, 2008 ▶); molecular graphics: *DIAMOND* (Brandenburg, 1999 ▶); software used to prepare material for publication: *SHELXL97*.

## Supplementary Material

Crystal structure: contains datablock(s) I, global. DOI: 10.1107/S1600536811042735/vn2018sup1.cif

Supplementary material file. DOI: 10.1107/S1600536811042735/vn2018Isup2.cdx

Structure factors: contains datablock(s) I. DOI: 10.1107/S1600536811042735/vn2018Isup3.hkl

Additional supplementary materials:  crystallographic information; 3D view; checkCIF report

## Figures and Tables

**Table 1:** Selected bond lengths (Å)

Eu1—O8	2.368 (4)
Eu1—O4	2.368 (3)
Eu1—O7	2.404 (4)
Eu1—O2	2.416 (3)
Eu1—O3	2.416 (3)
Eu1—O5	2.420 (4)
Eu1—O6	2.500 (3)
Eu1—O1	2.584 (4)
Eu1—O4^i^	2.889 (4)

Symmetry code: (i) 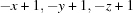.

**Table 2:** Hydrogen-bond geometry (Å, °)

*D*—H⋯*A*	*D*—H	H⋯*A*	*D*⋯*A*	*D*—H⋯*A*
O7—H7*B*⋯O6^ii^	0.86 (2)	1.90 (2)	2.756 (5)	174 (9)
O7—H7*A*⋯O2^i^	0.86 (2)	1.90 (4)	2.724 (5)	159 (9)

Symmetry codes: (i) 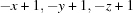; (ii) 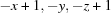.

**Fig. 1.**
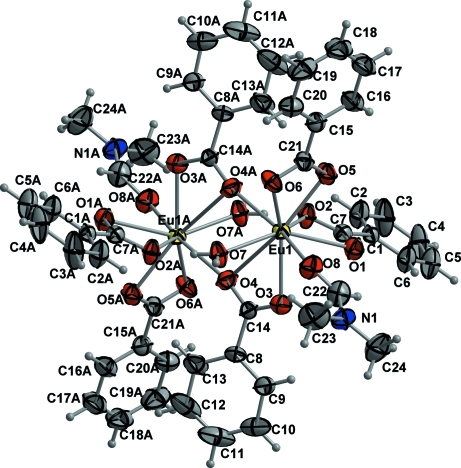
The molecular structure of (I), with atom labels and 50% probability displacement ellipsoids for non-H atoms. The suffix A corresponds to the symmetry code [-x + 1, -y + 1, -z + 1].

**Fig. 2.**
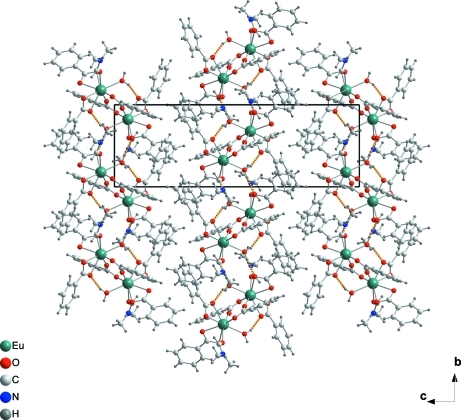
The packing of (I), viewed along the a axis. C—H···O hydrogen bonds are shown as yellow dashed lines in bold.

## supplementary crystallographic information

### Comment

Because of their excellent luminescent properties (Chin *et al.*, 1994; Singh *et al.*, 2002), rare earth–carboxylic acid complexes have been widely studied and applied in many fields. Distinct structure features with various rare earths (Qiu *et al.*, 2007; Gubina *et al.*, 2000) or ligands (Jin *et al.*, 1996; Wang *et al.*, 2003) have been reported. The title compound, (I) was synthesized and its structure was determined. Similar crystal structure with benzoate as ligands have been reported recently (Ooi *et al.*, 2010).

The centrosymmetric structure of the title complex (I) is shown in Fig. 1. The two Eu^3+^ ions are bridged by two tridentate bridging carboxylates. Each of the two Eu^3+^ ions is further coordinated by two chelating carboxlates, one DMF molecule and one water molecule, with an overall coordination number of nine. Eu—O bond distances are presented in Table 1.

In the crystal structure, intermolecular O7—H7B···O6^ii^ hydrogen bonds (Table 2) link molecules into chains along the *b* axis (Fig. 2). The molecular structure is stabilized by intramolecular O7—H7A···O2^i^ hydrogen bonds (Table 2).

### Experimental

0.5 g Eu_2_O_3_ (Strem, 99.99%) was dissolved in 5 ml 37% HCl and the solution was evaporated to dryness. 10 ml water was added to the residue and the pH was adjusted to 8 by the addition of NH_3_ (aq). The ensuing precipitate was filtered, washed with water and dried. This precipitate was added to a solution containing 1 g C_6_H_5_COOH and 20 ml DMF with stirring. The mixture was vigorously stirred and filtered. The filtrate was put in a beaker covered with parafilm and left in a dark fume cupboard at room temperature to form colorless needle crystals.

### Refinement

All aromatic and methyl H atoms were positioned geometrically with C—H = 0.93 and 0.96 Å, and were constrained to ride on their parent C atoms with *U*_iso_(H) = 1.2*U*_eq_(C) and 1.5*U*_eq_(C), respectively. The DANG and DFIX restraints were used in order to keep the geometry of the water molecule reasonable.

### Crystal data

**Table:**

[Eu_2_(C_7_H_5_O_2_)_6_(C_3_H_7_NO)_2_(H_2_O)_2_]	*F*(000) = 1208
*M**_r_* = 1212.80	*D*_x_ = 1.653 Mg m^−^^3^
Monoclinic, *P*2_1_/*c*	Cu *K*α radiation, λ = 1.54184 Å
Hall symbol: -P 2ybc	Cell parameters from 5244 reflections
*a* = 11.6395 (2) Å	θ = 3.5–67.5°
*b* = 8.3692 (1) Å	µ = 18.84 mm^−^^1^
*c* = 25.5235 (4) Å	*T* = 291 K
β = 101.460 (2)°	Block, colourless
*V* = 2436.76 (6) Å^3^	0.36 × 0.32 × 0.32 mm
*Z* = 2	

### Data collection

**Table:**

Oxford Gemini S Ultra diffractometer	4294 independent reflections
Radiation source: Enhance Ultra (Cu) X-ray source	3860 reflections with *I* > 2σ(*I*)
mirror	*R*_int_ = 0.035
Detector resolution: 15.9149 pixels mm^-1^	θ_max_ = 67.6°, θ_min_ = 3.5°
ω scans	*h* = −13→9
Absorption correction: multi-scan (*CrysAlis PRO*; Oxford Diffraction, 2010)	*k* = −9→5
*T*_min_ = 0.056, *T*_max_ = 0.065	*l* = −30→30
8782 measured reflections	

### Refinement

**Table:**

Refinement on *F*^2^	Primary atom site location: structure-invariant direct methods
Least-squares matrix: full	Secondary atom site location: difference Fourier map
*R*[*F*^2^ > 2σ(*F*^2^)] = 0.045	Hydrogen site location: inferred from neighbouring sites
*wR*(*F*^2^) = 0.120	H atoms treated by a mixture of independent and constrained refinement
*S* = 1.03	*w* = 1/[σ^2^(*F*_o_^2^) + (0.0781*P*)^2^] where *P* = (*F*_o_^2^ + 2*F*_c_^2^)/3
4294 reflections	(Δ/σ)_max_ = 0.003
315 parameters	Δρ_max_ = 1.20 e Å^−^^3^
3 restraints	Δρ_min_ = −1.92 e Å^−^^3^

### Special details

**Table:**

Experimental. CrysAlisPro (Oxford Diffraction, 2010). Empirical absorption correction using spherical harmonics, implemented in SCALE3 ABSPACK scaling algorithm.
Geometry. All e.s.d.'s (except the e.s.d. in the dihedral angle between two l.s. planes) are estimated using the full covariance matrix. The cell e.s.d.'s are taken into account individually in the estimation of e.s.d.'s in distances, angles and torsion angles; correlations between e.s.d.'s in cell parameters are only used when they are defined by crystal symmetry. An approximate (isotropic) treatment of cell e.s.d.'s is used for estimating e.s.d.'s involving l.s. planes.
Refinement. Refinement of *F*^2^ against ALL reflections. The weighted *R*-factor *wR* and goodness of fit *S* are based on *F*^2^, conventional *R*-factors *R* are based on *F*, with *F* set to zero for negative *F*^2^. The threshold expression of *F*^2^ > σ(*F*^2^) is used only for calculating *R*-factors(gt) *etc*. and is not relevant to the choice of reflections for refinement. *R*-factors based on *F*^2^ are statistically about twice as large as those based on *F*, and *R*-factors based on ALL data will be even larger.

### Fractional atomic coordinates and isotropic or equivalent isotropic displacement parameters (Å^2^)

**Table:**

	*x*	*y*	*z*	*U*_iso_*/*U*_eq_	
Eu1	0.58936 (2)	0.32204 (3)	0.555114 (10)	0.02985 (13)	
O1	0.7648 (3)	0.4223 (4)	0.62602 (15)	0.0471 (8)	
O5	0.5539 (3)	0.2337 (5)	0.64080 (14)	0.0455 (8)	
O8	0.7148 (3)	0.0976 (5)	0.55948 (16)	0.0499 (9)	
O2	0.6119 (3)	0.5789 (4)	0.59914 (14)	0.0426 (8)	
O7	0.5164 (4)	0.2066 (5)	0.46868 (15)	0.0460 (8)	
O4	0.4078 (3)	0.4576 (4)	0.53174 (15)	0.0450 (8)	
O6	0.4632 (3)	0.0941 (4)	0.57245 (14)	0.0442 (8)	
O3	0.7419 (3)	0.4010 (5)	0.50855 (15)	0.0530 (10)	
C1	0.7736 (4)	0.6905 (6)	0.65945 (19)	0.0347 (11)	
N1	0.8798 (4)	−0.0405 (6)	0.5577 (2)	0.0521 (11)	
C8	0.7618 (4)	0.5287 (6)	0.42852 (19)	0.0379 (10)	
C15	0.4233 (4)	0.0286 (6)	0.6587 (2)	0.0414 (11)	
C14	0.6945 (4)	0.4870 (6)	0.47094 (19)	0.0366 (10)	
C16	0.4494 (5)	0.0601 (8)	0.7132 (2)	0.0546 (14)	
H16	0.5050	0.1374	0.7265	0.065*	
C9	0.8819 (5)	0.5075 (8)	0.4393 (2)	0.0503 (14)	
H9	0.9175	0.4610	0.4716	0.060*	
C22	0.8222 (5)	0.0822 (7)	0.5720 (3)	0.0530 (13)	
H22	0.8650	0.1618	0.5926	0.064*	
C21	0.4839 (4)	0.1220 (6)	0.62194 (19)	0.0378 (10)	
C20	0.3412 (5)	−0.0855 (8)	0.6391 (3)	0.0540 (14)	
H20	0.3239	−0.1083	0.6027	0.065*	
C6	0.8917 (6)	0.6821 (7)	0.6827 (3)	0.0544 (16)	
H6	0.9329	0.5878	0.6808	0.065*	
C7	0.7149 (4)	0.5550 (6)	0.62677 (18)	0.0366 (10)	
C10	0.9505 (6)	0.5531 (9)	0.4038 (3)	0.0681 (19)	
H10	1.0315	0.5405	0.4122	0.082*	
C19	0.2840 (6)	−0.1669 (8)	0.6742 (4)	0.070 (2)	
H19	0.2274	−0.2430	0.6611	0.084*	
C18	0.3111 (6)	−0.1349 (10)	0.7283 (3)	0.070 (2)	
H18	0.2733	−0.1902	0.7516	0.084*	
C13	0.7090 (6)	0.5896 (8)	0.3796 (2)	0.0622 (17)	
H13	0.6281	0.6020	0.3713	0.075*	
C17	0.3936 (6)	−0.0219 (9)	0.7476 (3)	0.0668 (18)	
H17	0.4120	−0.0005	0.7841	0.080*	
C24	1.0054 (6)	−0.0566 (11)	0.5768 (4)	0.088 (3)	
H24B	1.0207	−0.1451	0.6011	0.132*	
H24A	1.0358	0.0396	0.5949	0.132*	
H24C	1.0428	−0.0750	0.5470	0.132*	
C5	0.9487 (7)	0.8130 (9)	0.7088 (3)	0.071 (2)	
H5	1.0283	0.8074	0.7237	0.085*	
C4	0.8876 (7)	0.9514 (9)	0.7128 (3)	0.077 (2)	
H4	0.9261	1.0399	0.7300	0.093*	
C12	0.7762 (8)	0.6330 (10)	0.3423 (3)	0.085 (3)	
H12	0.7403	0.6717	0.3089	0.102*	
C23	0.8170 (8)	−0.1668 (8)	0.5251 (4)	0.078 (2)	
H23B	0.8175	−0.2616	0.5463	0.118*	
H23A	0.8545	−0.1886	0.4956	0.118*	
H23C	0.7376	−0.1339	0.5119	0.118*	
C2	0.7126 (6)	0.8298 (7)	0.6634 (3)	0.0581 (16)	
H2	0.6336	0.8371	0.6475	0.070*	
C11	0.8975 (8)	0.6176 (10)	0.3556 (3)	0.082 (2)	
H11	0.9431	0.6514	0.3317	0.098*	
C3	0.7697 (7)	0.9596 (8)	0.6914 (3)	0.078 (2)	
H3	0.7280	1.0519	0.6955	0.093*	
H7B	0.527 (8)	0.112 (5)	0.458 (4)	0.117*	
H7A	0.470 (7)	0.253 (10)	0.443 (3)	0.117*	

### Atomic displacement parameters (Å^2^)

**Table:**

	*U*^11^	*U*^22^	*U*^33^	*U*^12^	*U*^13^	*U*^23^
Eu1	0.03041 (19)	0.0315 (2)	0.02777 (18)	0.00101 (9)	0.00612 (12)	−0.00123 (9)
O1	0.0428 (19)	0.048 (2)	0.048 (2)	0.0050 (16)	0.0030 (15)	−0.0044 (17)
O5	0.050 (2)	0.047 (2)	0.0392 (18)	−0.0082 (17)	0.0086 (15)	−0.0080 (17)
O8	0.050 (2)	0.042 (2)	0.057 (2)	0.0100 (17)	0.0084 (17)	−0.0019 (18)
O2	0.0427 (18)	0.0357 (19)	0.0443 (18)	0.0032 (14)	−0.0039 (14)	−0.0070 (15)
O7	0.061 (2)	0.0381 (19)	0.0350 (18)	0.0043 (18)	−0.0003 (16)	−0.0049 (15)
O4	0.0327 (17)	0.053 (2)	0.051 (2)	0.0026 (15)	0.0118 (15)	−0.0110 (17)
O6	0.060 (2)	0.0393 (19)	0.0350 (18)	−0.0067 (16)	0.0138 (16)	0.0023 (15)
O3	0.0423 (19)	0.073 (3)	0.046 (2)	0.0038 (19)	0.0142 (16)	0.012 (2)
C1	0.037 (3)	0.040 (3)	0.025 (2)	0.0001 (19)	0.0017 (19)	−0.0030 (18)
N1	0.048 (2)	0.048 (3)	0.061 (3)	0.009 (2)	0.013 (2)	0.002 (2)
C8	0.037 (2)	0.045 (3)	0.035 (2)	0.004 (2)	0.0158 (19)	−0.004 (2)
C15	0.039 (3)	0.041 (3)	0.046 (3)	0.006 (2)	0.012 (2)	0.011 (2)
C14	0.029 (2)	0.044 (3)	0.038 (2)	−0.003 (2)	0.0085 (19)	−0.006 (2)
C16	0.064 (3)	0.062 (4)	0.040 (3)	0.000 (3)	0.015 (3)	0.006 (3)
C9	0.038 (3)	0.067 (4)	0.049 (3)	−0.004 (3)	0.016 (2)	−0.008 (3)
C22	0.055 (3)	0.044 (3)	0.058 (3)	−0.001 (3)	0.005 (3)	−0.001 (3)
C21	0.040 (3)	0.038 (3)	0.036 (2)	0.006 (2)	0.010 (2)	0.006 (2)
C20	0.052 (3)	0.058 (4)	0.054 (3)	−0.005 (3)	0.017 (3)	0.004 (3)
C6	0.049 (3)	0.058 (4)	0.049 (3)	0.005 (2)	−0.010 (3)	−0.010 (3)
C7	0.036 (2)	0.044 (3)	0.030 (2)	0.004 (2)	0.0063 (18)	−0.001 (2)
C10	0.059 (4)	0.078 (5)	0.078 (5)	−0.005 (3)	0.040 (4)	−0.005 (4)
C19	0.056 (4)	0.064 (4)	0.093 (6)	−0.011 (3)	0.022 (4)	0.019 (4)
C18	0.063 (4)	0.080 (5)	0.074 (5)	0.010 (4)	0.029 (4)	0.032 (4)
C13	0.069 (4)	0.076 (4)	0.046 (3)	0.036 (3)	0.022 (3)	0.012 (3)
C17	0.079 (4)	0.079 (5)	0.048 (3)	0.008 (4)	0.026 (3)	0.018 (3)
C24	0.061 (4)	0.100 (6)	0.101 (6)	0.029 (4)	0.011 (4)	0.014 (5)
C5	0.060 (4)	0.078 (5)	0.063 (4)	−0.008 (3)	−0.014 (3)	−0.008 (3)
C4	0.093 (5)	0.060 (4)	0.062 (4)	−0.017 (4)	−0.026 (4)	−0.009 (3)
C12	0.128 (7)	0.085 (5)	0.054 (4)	0.053 (5)	0.047 (4)	0.025 (4)
C23	0.083 (5)	0.060 (4)	0.095 (6)	0.006 (3)	0.024 (5)	−0.022 (4)
C2	0.054 (4)	0.059 (4)	0.053 (4)	0.008 (3)	−0.010 (3)	−0.013 (3)
C11	0.107 (6)	0.068 (5)	0.091 (6)	0.018 (4)	0.072 (5)	0.016 (4)
C3	0.099 (5)	0.051 (4)	0.066 (4)	0.012 (4)	−0.026 (4)	−0.027 (3)

### Geometric parameters (Å, °)

**Table:**

Eu1—O8	2.368 (4)	C16—C17	1.374 (8)
Eu1—O4	2.368 (3)	C16—H16	0.9300
Eu1—O7	2.404 (4)	C9—C10	1.377 (8)
Eu1—O2	2.416 (3)	C9—H9	0.9300
Eu1—O3	2.416 (3)	C22—H22	0.9300
Eu1—O5	2.420 (4)	C20—C19	1.395 (9)
Eu1—O6	2.500 (3)	C20—H20	0.9300
Eu1—O1	2.584 (4)	C6—C5	1.380 (10)
Eu1—C21	2.837 (5)	C6—H6	0.9300
Eu1—C7	2.867 (5)	C10—C11	1.371 (11)
Eu1—O4^i^	2.889 (4)	C10—H10	0.9300
Eu1—C14	3.011 (5)	C19—C18	1.380 (12)
O1—C7	1.255 (6)	C19—H19	0.9300
O5—C21	1.271 (6)	C18—C17	1.368 (11)
O8—C22	1.235 (7)	C18—H18	0.9300
O2—C7	1.280 (6)	C13—C12	1.394 (9)
O7—H7B	0.86 (2)	C13—H13	0.9300
O7—H7A	0.86 (2)	C17—H17	0.9300
O4—C14^i^	1.267 (5)	C24—H24B	0.9600
O4—Eu1^i^	2.889 (4)	C24—H24A	0.9600
O6—C21	1.260 (6)	C24—H24C	0.9600
O3—C14	1.238 (6)	C5—C4	1.373 (10)
C1—C2	1.379 (8)	C5—H5	0.9300
C1—C6	1.388 (8)	C4—C3	1.374 (10)
C1—C7	1.490 (7)	C4—H4	0.9300
N1—C22	1.316 (7)	C12—C11	1.392 (12)
N1—C23	1.450 (9)	C12—H12	0.9300
N1—C24	1.453 (8)	C23—H23B	0.9600
C8—C13	1.376 (8)	C23—H23A	0.9600
C8—C9	1.382 (7)	C23—H23C	0.9600
C8—C14	1.498 (6)	C2—C3	1.393 (9)
C15—C20	1.373 (8)	C2—H2	0.9300
C15—C16	1.388 (8)	C11—H11	0.9300
C15—C21	1.501 (7)	C3—H3	0.9300
C14—O4^i^	1.267 (5)		
			
O8—Eu1—O4	154.52 (13)	C13—C8—C9	118.7 (5)
O8—Eu1—O7	80.01 (13)	C13—C8—C14	122.6 (5)
O4—Eu1—O7	80.00 (13)	C9—C8—C14	118.6 (5)
O8—Eu1—O2	132.46 (13)	C20—C15—C16	119.7 (5)
O4—Eu1—O2	72.54 (12)	C20—C15—C21	120.9 (5)
O7—Eu1—O2	140.39 (13)	C16—C15—C21	119.4 (5)
O8—Eu1—O3	74.58 (14)	O3—C14—O4^i^	121.6 (4)
O4—Eu1—O3	116.81 (13)	O3—C14—C8	118.7 (4)
O7—Eu1—O3	79.55 (15)	O4^i^—C14—C8	119.8 (4)
O2—Eu1—O3	87.86 (14)	O3—C14—Eu1	50.3 (2)
O8—Eu1—O5	85.94 (14)	O4^i^—C14—Eu1	72.3 (3)
O4—Eu1—O5	94.03 (13)	C8—C14—Eu1	165.4 (3)
O7—Eu1—O5	127.89 (13)	C17—C16—C15	120.5 (6)
O2—Eu1—O5	82.75 (13)	C17—C16—H16	119.7
O3—Eu1—O5	143.29 (13)	C15—C16—H16	119.7
O8—Eu1—O6	76.39 (14)	C10—C9—C8	121.9 (6)
O4—Eu1—O6	83.33 (13)	C10—C9—H9	119.0
O7—Eu1—O6	75.10 (13)	C8—C9—H9	119.0
O2—Eu1—O6	127.60 (12)	O8—C22—N1	123.7 (6)
O3—Eu1—O6	144.16 (14)	O8—C22—H22	118.2
O5—Eu1—O6	52.81 (12)	N1—C22—H22	118.2
O8—Eu1—O1	80.26 (13)	O6—C21—O5	119.8 (4)
O4—Eu1—O1	124.21 (12)	O6—C21—C15	120.7 (5)
O7—Eu1—O1	149.22 (13)	O5—C21—C15	119.5 (4)
O2—Eu1—O1	52.24 (11)	O6—C21—Eu1	61.7 (3)
O3—Eu1—O1	72.56 (13)	O5—C21—Eu1	58.1 (2)
O5—Eu1—O1	73.68 (12)	C15—C21—Eu1	175.2 (4)
O6—Eu1—O1	122.27 (12)	C15—C20—C19	119.5 (6)
O8—Eu1—C21	80.78 (14)	C15—C20—H20	120.3
O4—Eu1—C21	87.89 (13)	C19—C20—H20	120.3
O7—Eu1—C21	101.45 (15)	C5—C6—C1	120.5 (6)
O2—Eu1—C21	105.40 (14)	C5—C6—H6	119.8
O3—Eu1—C21	154.81 (14)	C1—C6—H6	119.8
O5—Eu1—C21	26.48 (14)	O1—C7—O2	121.0 (5)
O6—Eu1—C21	26.34 (13)	O1—C7—C1	121.3 (4)
O1—Eu1—C21	98.42 (13)	O2—C7—C1	117.7 (4)
O8—Eu1—C7	106.16 (14)	O1—C7—Eu1	64.3 (3)
O4—Eu1—C7	98.69 (13)	O2—C7—Eu1	56.8 (2)
O7—Eu1—C7	154.32 (14)	C1—C7—Eu1	173.3 (3)
O2—Eu1—C7	26.30 (12)	C11—C10—C9	118.9 (7)
O3—Eu1—C7	78.27 (14)	C11—C10—H10	120.5
O5—Eu1—C7	77.76 (13)	C9—C10—H10	120.5
O6—Eu1—C7	130.46 (12)	C18—C19—C20	120.3 (7)
O1—Eu1—C7	25.97 (12)	C18—C19—H19	119.9
C21—Eu1—C7	104.14 (14)	C20—C19—H19	119.9
O8—Eu1—O4^i^	116.21 (12)	C17—C18—C19	119.9 (6)
O4—Eu1—O4^i^	69.30 (12)	C17—C18—H18	120.0
O7—Eu1—O4^i^	66.92 (12)	C19—C18—H18	120.0
O2—Eu1—O4^i^	76.82 (11)	C8—C13—C12	120.5 (6)
O3—Eu1—O4^i^	47.66 (11)	C8—C13—H13	119.8
O5—Eu1—O4^i^	156.69 (11)	C12—C13—H13	119.8
O6—Eu1—O4^i^	136.02 (11)	C18—C17—C16	120.1 (7)
O1—Eu1—O4^i^	101.67 (11)	C18—C17—H17	119.9
C21—Eu1—O4^i^	155.54 (12)	C16—C17—H17	119.9
C7—Eu1—O4^i^	88.47 (12)	N1—C24—H24B	109.5
O8—Eu1—C14	93.62 (13)	N1—C24—H24A	109.5
O4—Eu1—C14	93.99 (13)	H24B—C24—H24A	109.5
O7—Eu1—C14	69.44 (14)	N1—C24—H24C	109.5
O2—Eu1—C14	84.36 (13)	H24B—C24—H24C	109.5
O3—Eu1—C14	23.22 (13)	H24A—C24—H24C	109.5
O5—Eu1—C14	162.04 (12)	C4—C5—C6	119.9 (7)
O6—Eu1—C14	144.35 (12)	C4—C5—H5	120.1
O1—Eu1—C14	88.52 (12)	C6—C5—H5	120.1
C21—Eu1—C14	170.15 (14)	C5—C4—C3	120.2 (6)
C7—Eu1—C14	85.15 (13)	C5—C4—H4	119.9
O4^i^—Eu1—C14	24.70 (11)	C3—C4—H4	119.9
C7—O1—Eu1	89.7 (3)	C11—C12—C13	119.2 (7)
C21—O5—Eu1	95.4 (3)	C11—C12—H12	120.4
C22—O8—Eu1	132.7 (4)	C13—C12—H12	120.4
C7—O2—Eu1	96.9 (3)	N1—C23—H23B	109.5
Eu1—O7—H7B	128 (6)	N1—C23—H23A	109.5
Eu1—O7—H7A	126 (6)	H23B—C23—H23A	109.5
H7B—O7—H7A	106 (5)	N1—C23—H23C	109.5
C14^i^—O4—Eu1	166.2 (3)	H23B—C23—H23C	109.5
C14^i^—O4—Eu1^i^	83.0 (3)	H23A—C23—H23C	109.5
Eu1—O4—Eu1^i^	110.70 (12)	C1—C2—C3	119.8 (6)
C21—O6—Eu1	92.0 (3)	C1—C2—H2	120.1
C14—O3—Eu1	106.4 (3)	C3—C2—H2	120.1
C2—C1—C6	119.4 (5)	C10—C11—C12	120.7 (6)
C2—C1—C7	119.9 (5)	C10—C11—H11	119.7
C6—C1—C7	120.5 (5)	C12—C11—H11	119.7
C22—N1—C23	120.1 (5)	C4—C3—C2	120.1 (7)
C22—N1—C24	120.9 (6)	C4—C3—H3	119.9
C23—N1—C24	118.9 (6)	C2—C3—H3	119.9
			
O8—Eu1—O1—C7	175.9 (3)	O8—Eu1—C14—O4^i^	157.2 (3)
O4—Eu1—O1—C7	−11.9 (3)	O4—Eu1—C14—O4^i^	1.5 (3)
O7—Eu1—O1—C7	125.1 (3)	O7—Eu1—C14—O4^i^	79.3 (3)
O2—Eu1—O1—C7	−2.1 (3)	O2—Eu1—C14—O4^i^	−70.5 (3)
O3—Eu1—O1—C7	99.1 (3)	O3—Eu1—C14—O4^i^	−168.3 (5)
O5—Eu1—O1—C7	−95.5 (3)	O5—Eu1—C14—O4^i^	−114.8 (4)
O6—Eu1—O1—C7	−117.1 (3)	O6—Eu1—C14—O4^i^	85.6 (3)
C21—Eu1—O1—C7	−105.1 (3)	O1—Eu1—C14—O4^i^	−122.7 (3)
O4^i^—Eu1—O1—C7	60.9 (3)	C7—Eu1—C14—O4^i^	−96.9 (3)
C14—Eu1—O1—C7	81.9 (3)	O8—Eu1—C14—C8	10.2 (13)
O8—Eu1—O5—C21	−77.4 (3)	O4—Eu1—C14—C8	−145.4 (13)
O4—Eu1—O5—C21	77.0 (3)	O7—Eu1—C14—C8	−67.7 (13)
O7—Eu1—O5—C21	−3.4 (4)	O2—Eu1—C14—C8	142.6 (13)
O2—Eu1—O5—C21	148.8 (3)	O3—Eu1—C14—C8	44.8 (12)
O3—Eu1—O5—C21	−134.7 (3)	O5—Eu1—C14—C8	98.2 (13)
O6—Eu1—O5—C21	−1.4 (3)	O6—Eu1—C14—C8	−61.3 (13)
O1—Eu1—O5—C21	−158.4 (3)	O1—Eu1—C14—C8	90.4 (13)
C7—Eu1—O5—C21	175.1 (3)	C7—Eu1—C14—C8	116.2 (13)
O4^i^—Eu1—O5—C21	120.0 (3)	O4^i^—Eu1—C14—C8	−146.9 (14)
C14—Eu1—O5—C21	−166.6 (4)	C20—C15—C16—C17	0.0 (9)
O4—Eu1—O8—C22	169.1 (5)	C21—C15—C16—C17	178.9 (5)
O7—Eu1—O8—C22	130.3 (5)	C13—C8—C9—C10	3.4 (10)
O2—Eu1—O8—C22	−23.8 (6)	C14—C8—C9—C10	−175.5 (6)
O3—Eu1—O8—C22	48.5 (5)	Eu1—O8—C22—N1	−159.4 (4)
O5—Eu1—O8—C22	−100.1 (5)	C23—N1—C22—O8	0.5 (10)
O6—Eu1—O8—C22	−152.8 (5)	C24—N1—C22—O8	−175.8 (6)
O1—Eu1—O8—C22	−26.0 (5)	Eu1—O6—C21—O5	−2.5 (5)
C21—Eu1—O8—C22	−126.2 (5)	Eu1—O6—C21—C15	175.2 (4)
C7—Eu1—O8—C22	−24.1 (5)	Eu1—O5—C21—O6	2.6 (5)
O4^i^—Eu1—O8—C22	72.3 (5)	Eu1—O5—C21—C15	−175.1 (4)
C14—Eu1—O8—C22	61.9 (5)	C20—C15—C21—O6	−2.2 (8)
O8—Eu1—O2—C7	−0.7 (4)	C16—C15—C21—O6	179.0 (5)
O4—Eu1—O2—C7	173.6 (3)	C20—C15—C21—O5	175.5 (5)
O7—Eu1—O2—C7	−138.2 (3)	C16—C15—C21—O5	−3.3 (8)
O3—Eu1—O2—C7	−67.4 (3)	O8—Eu1—C21—O6	−77.9 (3)
O5—Eu1—O2—C7	77.0 (3)	O4—Eu1—C21—O6	79.2 (3)
O6—Eu1—O2—C7	106.9 (3)	O7—Eu1—C21—O6	−0.2 (3)
O1—Eu1—O2—C7	2.1 (3)	O2—Eu1—C21—O6	150.4 (3)
C21—Eu1—O2—C7	90.8 (3)	O3—Eu1—C21—O6	−89.9 (4)
O4^i^—Eu1—O2—C7	−114.3 (3)	O5—Eu1—C21—O6	−177.4 (5)
C14—Eu1—O2—C7	−90.5 (3)	O1—Eu1—C21—O6	−156.5 (3)
O8—Eu1—O4—C14^i^	78.9 (14)	C7—Eu1—C21—O6	177.6 (3)
O7—Eu1—O4—C14^i^	117.7 (14)	O4^i^—Eu1—C21—O6	58.5 (5)
O2—Eu1—O4—C14^i^	−91.2 (14)	O8—Eu1—C21—O5	99.5 (3)
O3—Eu1—O4—C14^i^	−169.5 (14)	O4—Eu1—C21—O5	−103.4 (3)
O5—Eu1—O4—C14^i^	−10.1 (14)	O7—Eu1—C21—O5	177.2 (3)
O6—Eu1—O4—C14^i^	41.7 (14)	O2—Eu1—C21—O5	−32.2 (3)
O1—Eu1—O4—C14^i^	−83.1 (14)	O3—Eu1—C21—O5	87.5 (5)
C21—Eu1—O4—C14^i^	15.6 (14)	O6—Eu1—C21—O5	177.4 (5)
C7—Eu1—O4—C14^i^	−88.3 (14)	O1—Eu1—C21—O5	20.9 (3)
O4^i^—Eu1—O4—C14^i^	−173.4 (15)	C7—Eu1—C21—O5	−5.0 (3)
C14—Eu1—O4—C14^i^	−174.0 (13)	O4^i^—Eu1—C21—O5	−124.1 (3)
O8—Eu1—O4—Eu1^i^	−107.7 (3)	C16—C15—C20—C19	0.7 (9)
O7—Eu1—O4—Eu1^i^	−68.97 (14)	C21—C15—C20—C19	−178.1 (5)
O2—Eu1—O4—Eu1^i^	82.15 (14)	C2—C1—C6—C5	−1.3 (11)
O3—Eu1—O4—Eu1^i^	3.84 (19)	C7—C1—C6—C5	174.0 (7)
O5—Eu1—O4—Eu1^i^	163.24 (13)	Eu1—O1—C7—O2	3.6 (5)
O6—Eu1—O4—Eu1^i^	−144.95 (14)	Eu1—O1—C7—C1	−175.9 (4)
O1—Eu1—O4—Eu1^i^	90.26 (16)	Eu1—O2—C7—O1	−3.9 (5)
C21—Eu1—O4—Eu1^i^	−170.98 (15)	Eu1—O2—C7—C1	175.7 (4)
C7—Eu1—O4—Eu1^i^	85.03 (14)	C2—C1—C7—O1	−173.9 (5)
O4^i^—Eu1—O4—Eu1^i^	0.0	C6—C1—C7—O1	10.8 (8)
C14—Eu1—O4—Eu1^i^	−0.67 (15)	C2—C1—C7—O2	6.5 (7)
O8—Eu1—O6—C21	96.7 (3)	C6—C1—C7—O2	−168.7 (5)
O4—Eu1—O6—C21	−98.8 (3)	O8—Eu1—C7—O1	−4.2 (3)
O7—Eu1—O6—C21	179.8 (3)	O4—Eu1—C7—O1	170.1 (3)
O2—Eu1—O6—C21	−36.9 (3)	O7—Eu1—C7—O1	−104.9 (4)
O3—Eu1—O6—C21	133.4 (3)	O2—Eu1—C7—O1	176.3 (5)
O5—Eu1—O6—C21	1.5 (3)	O3—Eu1—C7—O1	−74.2 (3)
O1—Eu1—O6—C21	27.8 (3)	O5—Eu1—C7—O1	77.8 (3)
C7—Eu1—O6—C21	−3.0 (4)	O6—Eu1—C7—O1	81.5 (3)
O4^i^—Eu1—O6—C21	−149.5 (3)	C21—Eu1—C7—O1	80.1 (3)
C14—Eu1—O6—C21	173.7 (3)	O4^i^—Eu1—C7—O1	−121.1 (3)
O8—Eu1—O3—C14	144.0 (4)	C14—Eu1—C7—O1	−96.6 (3)
O4—Eu1—O3—C14	−11.5 (4)	O8—Eu1—C7—O2	179.5 (3)
O7—Eu1—O3—C14	61.6 (4)	O4—Eu1—C7—O2	−6.2 (3)
O2—Eu1—O3—C14	−80.7 (4)	O7—Eu1—C7—O2	78.8 (4)
O5—Eu1—O3—C14	−155.5 (3)	O3—Eu1—C7—O2	109.5 (3)
O6—Eu1—O3—C14	107.0 (4)	O5—Eu1—C7—O2	−98.5 (3)
O1—Eu1—O3—C14	−131.6 (4)	O6—Eu1—C7—O2	−94.8 (3)
C21—Eu1—O3—C14	156.3 (3)	O1—Eu1—C7—O2	−176.3 (5)
C7—Eu1—O3—C14	−105.4 (4)	C21—Eu1—C7—O2	−96.2 (3)
O4^i^—Eu1—O3—C14	−6.6 (3)	O4^i^—Eu1—C7—O2	62.6 (3)
Eu1—O3—C14—O4^i^	13.2 (6)	C14—Eu1—C7—O2	87.1 (3)
Eu1—O3—C14—C8	−168.3 (4)	C8—C9—C10—C11	−1.8 (11)
C13—C8—C14—O3	164.0 (6)	C15—C20—C19—C18	−1.1 (10)
C9—C8—C14—O3	−17.0 (8)	C20—C19—C18—C17	0.6 (11)
C13—C8—C14—O4^i^	−17.4 (8)	C9—C8—C13—C12	−1.7 (10)
C9—C8—C14—O4^i^	161.5 (5)	C14—C8—C13—C12	177.2 (6)
C13—C8—C14—Eu1	125.8 (12)	C19—C18—C17—C16	0.2 (11)
C9—C8—C14—Eu1	−55.2 (15)	C15—C16—C17—C18	−0.5 (10)
O8—Eu1—C14—O3	−34.6 (4)	C1—C6—C5—C4	1.4 (13)
O4—Eu1—C14—O3	169.7 (4)	C6—C5—C4—C3	0.7 (14)
O7—Eu1—C14—O3	−112.5 (4)	C8—C13—C12—C11	−1.6 (12)
O2—Eu1—C14—O3	97.8 (4)	C6—C1—C2—C3	−0.9 (10)
O5—Eu1—C14—O3	53.4 (6)	C7—C1—C2—C3	−176.2 (6)
O6—Eu1—C14—O3	−106.1 (4)	C9—C10—C11—C12	−1.6 (12)
O1—Eu1—C14—O3	45.6 (4)	C13—C12—C11—C10	3.3 (13)
C7—Eu1—C14—O3	71.3 (4)	C5—C4—C3—C2	−2.9 (13)
O4^i^—Eu1—C14—O3	168.3 (5)	C1—C2—C3—C4	3.0 (12)

Symmetry codes: (i) −*x*+1, −*y*+1, −*z*+1.

### Hydrogen-bond geometry (Å, °)

**Table:**

*D*—H···*A*	*D*—H	H···*A*	*D*···*A*	*D*—H···*A*
O7—H7B···O6^ii^	0.86 (2)	1.90 (2)	2.756 (5)	174 (9)
O7—H7A···O2^i^	0.86 (2)	1.90 (4)	2.724 (5)	159 (9)

Symmetry codes: (ii) −*x*+1, −*y*, −*z*+1; (i) −*x*+1, −*y*+1, −*z*+1.
